# Role of Brown Adipose Tissue in Adiposity Associated With Narcolepsy Type 1

**DOI:** 10.3389/fendo.2020.00145

**Published:** 2020-04-16

**Authors:** Maaike E. Straat, Mink S. Schinkelshoek, Rolf Fronczek, Gerrit Jan Lammers, Patrick C. N. Rensen, Mariëtte R. Boon

**Affiliations:** ^1^Division of Endocrinology, Department of Medicine, Leiden University Medical Center, Leiden, Netherlands; ^2^Einthoven Laboratory for Experimental Vascular Medicine, Leiden University Medical Center, Leiden, Netherlands; ^3^Department of Neurology, Leiden University Medical Center, Leiden, Netherlands; ^4^Sleep Wake Centre SEIN, Heemstede, Netherlands

**Keywords:** brown adipose tissue, energy metabolism, hypothalamus, narcolepsy type 1, obesity, orexin, sympathetic nervous system

## Abstract

Narcolepsy type 1 is a neurological sleep-wake disorder caused by the destruction of orexin (hypocretin)-producing neurons. These neurons are particularly located in the lateral hypothalamus and have widespread projections throughout the brain, where they are involved, e.g., in the regulation of the sleep-wake cycle and appetite. Interestingly, a higher prevalence of obesity has been reported in patients with narcolepsy type 1 compared to healthy controls, despite a normal to decreased food intake and comparable physical activity. This suggests the involvement of tissues implicated in total energy expenditure, including skeletal muscle, liver, white adipose tissue (WAT), and brown adipose tissue (BAT). Recent evidence from pre-clinical studies with orexin knock-out mice demonstrates a crucial role for the orexin system in the functionality of brown adipose tissue (BAT), probably through multiple pathways. Since BAT is a highly metabolically active organ that combusts fatty acids and glucose toward heat, thereby contributing to energy metabolism, this raises the question of whether BAT plays a role in the development of obesity and related metabolic diseases in narcolepsy type 1. BAT is densely innervated by the sympathetic nervous system that activates BAT, for instance, following cold exposure. The sympathetic outflow toward BAT is mainly mediated by the dorsomedial, ventromedial, arcuate, and paraventricular nuclei in the hypothalamus. This review focuses on the current knowledge on the role of the orexin system in the control of energy balance, with specific focus on BAT metabolism and adiposity in both preclinical and clinical studies.

## Introduction

Narcolepsy type 1 is a sleep-wake disorder characterized by excessive daytime sleepiness and episodes of sudden muscle weakness, known as cataplexy. Narcolepsy type 1 is caused by the loss of more than 90% of orexin-producing neurons in the hypothalamus (1). For several decades, a higher prevalence of obesity has been reported in patients with narcolepsy type 1 compared to healthy controls, despite a normal to decreased food intake (2, 3). However, the mechanism underlying the increased adiposity in this patient population remains unclear and may involve tissues implicated in total energy expenditure including skeletal muscle, liver, white adipose tissue, and brown adipose tissue (BAT). Indeed, results from preclinical studies suggest a role for BAT in the increased adiposity after disease onset. The purpose of this review is to give an overview of the current knowledge on the role of the orexin system in the control of energy balance including food intake and energy expenditure with special emphasis on BAT metabolism. The ultimate aim is to increase the knowledge on the pathophysiology of adiposity development in patients with narcolepsy type 1.

## Narcolepsy Type 1: Epidemiology and Pathophysiology

Narcolepsy type 1 is a rare neurological disorder characterized by a dysregulated sleep-wake cycle. Age of onset peaks in adolescence and the disease affects around 15–50 per 100,000 individuals in the United States and Europe. Whether gender differences in the prevalence of narcolepsy type 1 exist is still unclear, since several epidemiological studies in Europe and the United States show inconsistent results (4–6). Narcolepsy type 1 is caused by the destruction of orexin-producing neurons in the hypothalamus. The exact pathophysiology remains unclear, but it is presumably caused by multiple triggers, eventually leading to an autoimmune-mediated destruction of these neurons (1). Projections of orexin-producing neurons extend throughout the brain, where they, amongst others, are involved in the regulation of wakefulness, metabolic circuits, and autonomic function (1, 7, 8). The main symptoms of narcolepsy type 1 include excessive daytime sleepiness and cataplexy (1). Cataplexy is the sudden bilateral loss of muscle tone evoked by an emotional trigger, frequently after laughter. Consciousness is typically preserved in these attacks, which are mostly of short duration (9). Other symptoms of narcolepsy type 1 comprise, amongst others, sleep paralysis, hypnagogic and hypnopompic hallucinations, and fragmented night sleep (1).

## Adiposity in Narcolepsy Type 1

Interestingly, apart from symptoms associated with a dysregulated sleep-wake cycle, obesity, defined as a body mass index above 30 kg/m^2^, is significantly more prevalent in patients with narcolepsy type 1 compared with the general population. Already in 1934, an increased prevalence of obesity amongst patients with narcolepsy type 1 was reported (2). In the following decades, the BMI of patients with narcolepsy type 1 was repeatedly confirmed to be higher compared to the general population (10–14). It is estimated that obesity affects around 30% of patients with narcolepsy type 1, without statistically proven differences between men and women (11–13). A study performed in France even reported rates over 50% in children with narcolepsy type 1 (14). In comparison, the prevalence of obesity in the European Union was 14.9% in 2017 (15). Studies comparing patients with narcolepsy type 1 with patients who are diagnosed with idiopathic hypersomnia, a neurologic disorder characterized by excessive daytime sleepiness but with normal orexin signaling, found that patients with narcolepsy type 1 have a significantly higher BMI and a larger waist circumference compared with patients with idiopathic hypersomnia (12, 16). Moreover, children diagnosed with narcolepsy type 1 rapidly gain weight shortly after disease onset (17). This suggests a direct pathogenic link between a decreased orexin signaling and higher BMI in narcolepsy type 1, rather than disease-related behavior leading to weight gain. In order to understand the pathophysiological link between orexin and obesity, a further understanding of the orexin system is needed.

## The Orexin System: An Overview

Orexin A and orexin B, also known as hypocretin 1 and hypocretin 2, are neuropeptides discovered in 1998, by two independent research groups who both gave the peptides a different name (18, 19). The group who gave the neuropeptides the name “orexin,” which is derived from the Greek word for “appetite,”öρ*εξις*, recognized the orexin system as a regulator of the feeding system. This was due to the finding that the orexin-producing neurons are located in the lateral hypothalamic area (LHA), which was known as the main regulator of the feeding system (18, 20). The name “hypocretin” highlights both the hypothalamic origin and morphologic resemblance to incretin hormones (19). The neuropeptides are synthesized from a precursor peptide, prepro-orexin, and are produced by orexin-producing neurons mainly located in the LHA but also in the perifornical area, dorsomedial hypothalamic area (DMH), and posterior hypothalamus (21). Both orexin neuropeptides are able to bind to two G-protein coupled receptors, orexin receptor type 1 (OXR1), and orexin receptor type 2 (OXR2). Orexin A, compared to orexin B, has a 10 times higher affinity for OXR1, and both orexins have the same affinity for OXR2 (18). The two types of orexins and receptors appear to have a partly overlapping and partly distinct function (22). Besides orexins, the orexin-producing neurons also release other neuromodulators, such as glutamate, dynorphin, and neuronal activity-regulated pentraxin (NARP) (23–25).

The orexin-producing neurons have extended projections throughout the central nervous system, where they fulfill multiple functions (8). As mentioned above, the orexin system is now well-acknowledged to play a role in the regulation of sleep and wakefulness since orexin deficiency causes narcolepsy type 1 in humans and animals (7, 26). Furthermore, orexins play an important part in other motivational behaviors such as the regulation of body weight, autonomic function, the reward system, emotion, memory, and stress (18, 27). For these purposes, efferent signaling via monoamine neurons, such as the noradrenergic neurons of the locus coeruleus, the serotonergic neurons of the raphe nuclei, the histaminergic neurons of the posterior hypothalamus, and the dopaminergic neurons of the ventral tegmental area, appears to be particularly important (1, 28).

Afferent signaling toward the orexin-producing neurons provides information about the environmental state and originates from several distinct brain regions. During wakefulness, the cholinergic system of the basal forebrain and emotional stimuli from the limbic system excite the orexin-producing neurons. During sleep, the GABAergic neurons of the preoptic area inhibit orexin-producing neurons. The serotonergic neurons of the raphe nuclei are involved in a negative feedback pathway, involved in both afferent and efferent signaling systems (29, 30). Furthermore, orexin-producing neurons receive information about energy homeostasis from the arcuate nucleus of the hypothalamus (ARC), which is particularly involved in food intake, and through several humeral factors and food-related cues, which will be discussed later in this review (31, 32).

In the following paragraphs, we will focus on the role of the orexin system in the regulation of body weight through modulation of energy intake and expenditure.

## The Orexin System and Food Intake

The first study that described the existence of orexin neuropeptides, produced by neurons in the “feeding center” of the LHA, showed that centrally administered orexin stimulates food intake in rats. Furthermore, the study reported an increase of prepro-orexin mRNA in fasted rats (18). This indicates that orexin-producing neurons are able to register the food status in order to increase food intake in times of fasting and thereby maintain energy homeostasis (18). In line with this, OXR antagonists and orexin antibodies inhibit food intake after central administration, and orexin-deficient mice show decreased food intake (33–35). The latter supports the physiological function of orexin in the regulation of feeding behavior in addition to the pharmacological role of administered orexin. From an evolutionary point of view, feeding behavior has a close interplay with arousal, locomotor activity, and reward mechanisms. In response to food deprivation, arousal pathways are activated, resulting in the increased motor activity, and wakefulness necessary for food-seeking. The orexin system is proposed to play a role in this regulation by evoking a correct behavior in response to nutrient deprivation. In line with this, intracerebroventricular injection of orexin A increases food intake during the light inactive phase in rats but not in the dark phase when rodents are normally already awake (36). Furthermore, orexin signaling positively correlates with wakefulness, and centrally administrated orexin significantly correlates with an increase in vigilance and motor activity (37). Mice lacking orexin-producing neurons or the orexin gene do not show an increase in arousal or locomotor activity in response to fasting, which confirms the physiological role of orexin in the activation of arousal pathways (38). Interestingly, Gonzalez et al. (39) found that upregulated orexin levels in response to fasting drop directly after sensing food, even before digestion. This suggests that orexin is useful in case of food deprivation, but its function is terminated by the action of eating, regardless of nutritional value (39).

In addition to the increase of arousal and vigilance to promote food intake, the orexin-producing neurons also more directly, and independently from arousal pathways, increase food consumption. They do so by projecting to other hypothalamic regions. For instance, orexins are found to have an excitatory effect on melanin-concentrating hormone (MCH) neurons, which anatomically also have a close relationship to orexin-producing neurons in the LHA (40). Furthermore, the orexins modulate orexigenic and anorexigenic neuron populations in the arcuate nucleus of the hypothalamus (ARC). Pro-opiomelanocortin (POMC) neurons in the ARC produce α-melanocyte-stimulating hormone (α-MSH), which stimulates melanocortin receptors in the paraventricular nucleus of the hypothalamus (PVN) to reduce food intake. Orexin A suppresses POMC neurons, leading to lower levels of α-MSH in mice and thereby possibly to hyperphagia (41). The strong orexigenic neuropeptide-Y (NPY) is also produced in the ARC and is bidirectionally involved in orexin signaling. Orexin-induced food intake partially runs via NPY neurons in the ARC, but in turn, NPY neurons modulate the orexin-producing neurons in the LHA as well (31, 42, 43).

**Figure 1:**
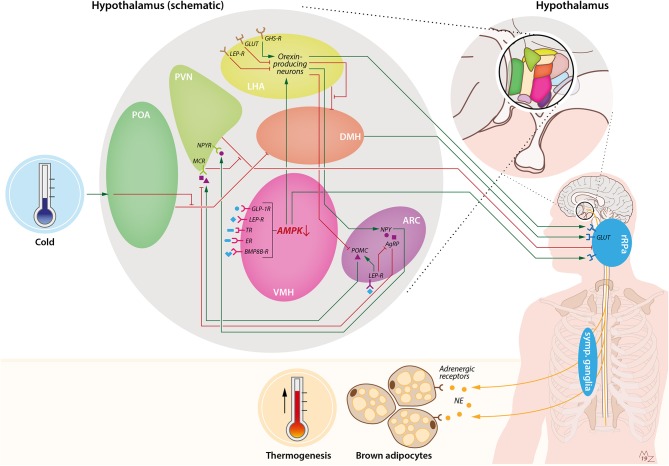
Hypothalamic pathways involved in sympathetic activation of BAT by cold and orexin. Cold exposure activates BAT by stimulation of the preoptic area of the hypothalamus (POA). This leads to suppression of the inhibiting GABAergic tone toward the dorsomedial nucleus of the hypothalamus (DMH), resulting in stimulation of rostral raphe pallidus (rRPa) neurons that are located in the medulla oblongata. From the rRPa, the activity of sympathetic neurons that run toward brown adipocytes is subsequently activated to release noradrenalin (NE), which binds to adrenergic receptors on brown adipocytes to induce thermogenesis. Several other brain pathways converge onto BAT. For instance, the ventromedial nucleus of the hypothalamus (VMH) possesses several receptors for peripheral signals such as GLP-1, leptin, thyroid hormone, estradiol, and bone morphogenetic protein 8B (BMP8B), which lead to activation of BAT via inhibition of AMP-activated protein kinase (AMPK) and stimulation of the rRPa. In addition, the arcuate nucleus of the hypothalamus (ARC) is involved in the thermoregulatory responses by BAT via the anorexigenic POMC neurons and orexigenic agouti-related peptide (AgRP) and neuropeptide-Y (NPY) neurons. Activation of pro-opiomelanocortin (POMC) neurons leads to stimulation of melanocortin receptors (MCRs) in the paraventricular nucleus of the hypothalamus (PVN), which in turn inhibits the GABAergic tone toward the rRPa. On the contrary, stimulation of AgRP neurons in the ARC results in inhibition of the MCR. NPY neurons in the ARC stimulate the neuropeptide-Y receptor (NPYR) on the PVN, thereby suppressing BAT thermogenesis through the GABAergic tone from the PVN toward the rRPa. Within the ARC, leptin stimulates POMC neurons and inhibits AgRP neurons, thereby stimulating BAT thermogenesis. Orexin is connected to BAT via several pathways. First, orexin-producing neurons, predominantly present in the lateral hypothalamic area (LHA), are able to inhibit the GABAergic tone of neurons in the LHA toward neurons in the DMH, thereby stimulating rRPa neurons. Furthermore, there is a link between the VMH and LHA via AMPK inhibition. Inhibition of AMPK in the VMH leads to stimulation of orexin-producing neurons and, subsequently, stimulation of rRPa neurons, resulting in sympathetic activation of BAT. Orexin-producing neurons also project to the ARC, where they inhibit POMC neurons and stimulate NPY neurons. Several food-related cues are able to influence orexin signaling. Glucose levels are negatively correlated with orexin signaling. Leptin is able to attenuate orexin-neuron activity, while ghrelin enhances orexin-neuron activity. BMP8B-R, bone morphogenetic protein 8B receptor; ER, estradiol receptor; GABA-R, GABA receptor; GHS-R, growth hormone secretagogue receptor (e.g., ghrelin receptor); GLP-1R, glucagon-like-peptide-1 receptor; GLUT, glucose transporter; LEP-R, leptin receptor; TR, thyroid receptor.

Various afferent modulating factors are known to stimulate orexin signaling (Figure 1). The stomach-derived hormone ghrelin, generally known to induce appetite in times of food restriction, activates orexin-producing neurons in the LHA (44). Correspondingly, glucose levels negatively influence orexin signaling, suggesting that low energy levels activate arousal pathways to provide energy homeostasis (38). In addition, the adipose tissue-derived hormone leptin, an appetite suppressor, attenuates orexin action, possibly through an indirect suppression via the adjacent neurotensin neurons (45, 46).

Thus, hypothalamic orexins are modulated by nutritional status to influence the regulation of food intake, doing so by the control of arousal and vigilance and by their effect on orexigenic and anti-orexigenic neuropeptides within the hypothalamus.

## Appetite in Narcolepsy Type 1

In line with the appetite-increasing effects of orexin, patients with narcolepsy type 1 tend to have a lower food intake compared to controls. Several studies have investigated whether differences in appetite hormones are related to this. The role of the adipokine leptin has been studied in several clinical trials with conflicting results. Smaller clinical cohort studies indicate lower plasma leptin levels and a loss of circadian rhythmicity in patients with narcolepsy type 1 (47, 48). However, the most recent and bigger cohort studies did not show a difference in leptin levels in plasma or cerebrospinal fluid, nor in circadian secretion of the hormone (49–51). The appetite-inducing hormone ghrelin seems not to be different between patients with narcolepsy type 1 and controls. Nonetheless, one study reports that plasma levels of the hormone obestatin, which is transcribed from the same gene as ghrelin, are elevated in narcolepsy type 1 patients compared to healthy controls (50, 52). This coincided with a disturbed autonomous function. The authors speculate that disturbed cholinergic signaling leads to higher obestatin release, but thus far, clear evidence for this hypothesis is lacking (52).

## Energy Expenditure in Narcolepsy Type 1

Despite a lower food intake, patients with narcolepsy type 1 tend to be overweight to obese (3, 10). Preclinical studies also reveal a higher fat accumulation in orexin-deficient rodents, despite a significantly lower calorie consumption (35). Obesity is the result of a disequilibrium between energy intake and energy expenditure. Therefore, orexins must also influence the other side of the energy balance, e.g., the regulation of energy expenditure. Orexin promotes food-seeking behavior, so it increases locomotor activity in rats (53). This would lead to the logical suggestion that in case of orexin deficiency, lower physical activity might contribute to the weight gain. However, in patients with narcolepsy type 1, a decrease in locomotor activity does not fully explain the amount of weight gain after disease onset (3, 54, 55). A lower metabolic rate is therefore hypothesized to be causally related to the positive energy balance in patients with narcolepsy type 1. Several studies in adolescents and adults demonstrated that basal metabolic rate did not significantly differ between patients with narcolepsy type 1 and healthy controls (54, 56, 57). In contrast, Dahmen et al. (57) found a significantly lower energy expenditure and basal metabolic rate in non-obese patients with narcolepsy type 1 compared with controls. This difference was not found in obese patients with narcolepsy type 1. The authors speculated that these findings could be explained by a mechanism in which the development of narcolepsy type 1 leads to a higher individual BMI set point that, in turn, leads to a decrease in basal metabolic rate. Once this BMI set point is reached, the metabolic rate will return to a normal level (57). In line with this, Wang et al. (58) showed that the BMI increase is higher in children diagnosed with narcolepsy type 1 compared to controls of the same age and that this BMI increase is accompanied by a decrease in basal metabolic rate. Both the higher increase in BMI and lower basal metabolic rate return to values observed in healthy age-matched controls 3–4 years after diagnosis (58).

Several metabolic organs contribute substantially to energy metabolism, including WAT, liver, muscle, and energy-combusting BAT. One previous study showed that patients with narcolepsy type 1 tend to have lower plasma glycerol levels compared to healthy controls, which might indicate a lower rate of lipolysis in adipose tissue, resulting in more fat storage. This coincided with higher insulin-induced glucose uptake by skeletal muscles, pointing toward more glycogenesis. Hepatic insulin sensitivity appeared to be unaffected (59). Thus, narcolepsy type 1 might result in a more “anabolic” state of the body. However, in-depth research into the underlying mechanism is missing. The sympathetic nervous system has a crucial role in the regulation of energy metabolism (60). Patients with narcolepsy type 1 show autonomic abnormalities, with some studies reporting a decreased sympathetic activity, heart rate variability, and blood pressure during wakefulness (56, 61). In contrast, those parameters are shown to be normal to high during sleep (62–64). However, standardized measurement methods are lacking, and untreated patients have an inability to remain awake, which makes reliable autonomic measurements a challenge (65). Nonetheless, several preclinical studies have revealed that the orexin system influences the sympathetic outflow toward peripheral tissues. More specifically, centrally administered orexin in rodents induces a rise in blood pressure, heart rate, and plasma catecholamine levels and increases in energy expenditure and thermogenesis (66–68). The increase in thermogenesis might be due to an increase in sympathetic outflow toward energy-combusting BAT, on which we will focus in the next paragraph.

## Activation of Brown Adipose Tissue

BAT is an organ that is particularly involved in non-shivering thermogenesis and is therewith known to significantly contribute to energy metabolism in rodents as well as in humans (69). After activation, BAT combusts triglyceride-derived fatty acids and glucose into heat due to the unique presence of uncoupling protein-1 (UCP-1) in the inner mitochondrial membrane. Morphologically, brown adipocytes contain numerous small lipid droplets and possess large numbers of mitochondria, resulting in the characteristic brownish color. Besides the classical brown adipocytes, which lie in depots around the cervical and supraclavicular area, brown-like adipocytes, so called “beige/brite” cells that are scattered within white adipose tissue, are also involved in the process of thermogenesis (70). In mice, prolonged activation of BAT through cold exposure or by directly targeting certain receptors present on BAT induces weight loss and reduces plasma triglycerides and cholesterol (71, 72). Consequently, this leads to a reduction of atherosclerosis development (71). In humans, activation of BAT by cold exposure increases energy metabolism and decreases fat mass, thereby being a promising target to combat adiposity (73). The current gold standard to visualize BAT in human is by measuring its glucose uptake with a [^18^F]fluorodeoxyglucose ([^18^F]FDG) positron emission tomography (PET)/computed tomography (CT) scan. This method uses the glucose analog [^18^F]FDG as a tracer to visualize glucose uptake by metabolically active tissues (74, 75).

BAT is known to be strongly innervated by the sympathetic nervous system, which activates BAT following a variety of stimuli, of which cold exposure is the best known (76). Cold exposure results in activation of receptors from the transient receptor potential (TRP) family in the skin. Then, these receptors send a signal via the dorsal horn neurons toward the preoptic area of the hypothalamus (POA; Figure 1) (77–79). From here, the efferent signal runs through the dorsomedial nucleus of the hypothalamus (DMH). In thermoneutral conditions, the DMH is inhibited through a continuous GABAergic tone from neurons in the POA. However, stimulation of the POA by a cold stimulus provokes inhibition of this GABAergic tone toward the DMH, resulting in stimulation of glutamate receptors at the medullary rostral raphe pallidus (rRPa) neurons. Sympathetic premotor neurons at the rRPa generate sympathetic outflow toward BAT, resulting in activation of BAT and enhanced thermogenesis (78, 80–82). Several additional efferent routes involving different hypothalamic nuclei and pathways are involved in the control of thermogenesis in response to various other stimuli. In general, all of these pathways lead to the generation of sympathetic outflow from BAT sympathetic premotor neurons at the medullary rRPa (80). One of those nuclei is the ventromedial nucleus of the hypothalamus (VMH) (83, 84). Preclinical studies show that several hormones influencing energy homeostasis modulate BAT activity independently of cold through the VMH. These hormones, such as thyroid hormones, bone morphogenetic protein 8B (BMP8B), glucagon-like-peptide-1, leptin, and estradiol, enhance the sympathetic outflow toward BAT via the rRPa after binding in the VMH. Multiple studies reveal that they do so by modulation of a pathway that involves inhibition of AMP-activated protein kinase (AMPK), which subsequently results in stimulation of sympathetic nerve fibers (80, 81, 85–90). Furthermore, neuron populations in the ARC play an important role in modulation of BAT activity (81, 91). Amongst those are the orexigenic NPY- and agouti-related peptide (AgRP)-expressing neurons and the anorexigenic POMC neurons (92). As mentioned before, the POMC neurons excrete α-MSH, which stimulates melanocortin receptors in the PVN. Apart from suppression of food intake, this also enhances sympathetic outflow toward BAT through inhibition of GABAergic projections to the rRPa, resulting in a negative energy balance (93). On the contrary, AgRP and NPY are thought to stimulate appetite and reduce sympathetic outflow toward BAT through inhibition of the melanocortin receptors and stimulation of the NPY receptors, respectively (80, 94). Leptin consecutively induces the POMC neurons, and thus α-MSH, to signal toward the melanocortin receptors in the PVN and thereby enhances thermogenesis through the same pathway. Simultaneously leptin inhibits the AgRP and NPY neurons (95).

In conclusion, BAT is a highly metabolically active organ with a function in thermoregulation. BAT is densely innervated by the sympathetic nervous system, and several hypothalamic nuclei and pathways are involved in the central stimulation of BAT. Interestingly, the neuronal areas and neuropeptides involved in the regulation of BAT partly overlap those involved in the orexin system. Both are acknowledged players in energy metabolism. Therefore, the following paragraph focuses on the interplay between the two systems.

## Role of the Orexin System in Brown Adipose Tissue Activation

Compelling evidence points toward a role of the orexin system in the regulation of thermogenesis by BAT. Yet, uncertainty exists about the exact mechanism and pathways involved. In 1999, increased plasma norepinephrine levels were shown after central injection of a high dose of orexin A but not orexin B in mice (66). After that, multiple studies followed that investigated the pharmacological effect of intracerebroventricular administration of orexin A, and the majority showed an increase in thermogenesis (67, 96–99). However, the involvement of BAT remained controversial due to contrasting results found by different research groups. In 2003, centrally injected orexin A was shown to increase whole-body energy expenditure, colonic temperature, and heart rate in rats, but the involvement of BAT was not specifically studied (67). Monda et al. (96–99) incorporated BAT in their studies and showed an increase in sympathetic firing rate toward BAT and a rise in BAT temperature after injection of orexin A in rats. On the contrary, another research group suggested that the thermogenic response upon orexin A is not regulated by BAT but rather by skeletal muscle. They demonstrated an increase in *Ucp3* mRNA expression in skeletal muscle after central orexin A administration, but no changes in *Ucp1* on BAT (100). In line with this, Haynes et al. (36) did not find temperature changes in BAT after 8 days of orexin A infusion in rats. Furthermore, in obese mice, they show that an orexin 1 receptor antagonist reduces weight gain and BAT weight and upregulates *Ucp1* expression (101). In lean rats, orexin 1 receptor antagonism also leads to an upregulation of *Ucp1* expression together with an increase in BAT thermogenesis and a reduction in body weight (102).

The discrepancy in results points toward a more complex involvement of the orexin system in the regulation of thermogenesis by BAT. Besides the orexin neuropeptides, other co-existent neurotransmitters are released by the orexin-producing neurons such as glutamate and dynorphin (23, 24). To address the difference between involvement of the orexin neuropeptides and the orexin-producing neurons, Zhang et al. (103) investigated the effect of an orexin neuropeptide knockout model (OX-KO) vs. the complete ablation of the orexin-producing neurons (OX-AB) in mice. OX-KO mice have a translocation in the prepro-orexin gene and, therefore, do not produce orexin neuropeptides, whereas OX-AB mice have completely ablated orexin-producing neurons and hence also lack the co-existent modulators in addition to orexin. The study demonstrated that OX-AB mice showed lower BAT activity and *Ucp1* expression in response to stress, while OX-KO mice did not show a difference in BAT activity (103), pointing toward the involvement of the orexin-producing neurons instead of the orexin neuropeptides. Several years later, the same group showed that the thermogenic fever response upon prostaglandin E_2_ (PGE_2_) injection in the medial POA was attenuated in OX-AB mice but not in OX-KO mice. OX-AB mice were less tolerant to cold exposure, despite similar locomotor activity compared to OX-KO and wildtype mice. BAT morphology appeared to be normal in both mouse models. In addition, they showed that treatment with the glutamate receptor antagonist prior to PGE_2_ injection or cold exposure inhibited a thermogenic response in wildtype mice (104). The involvement of the orexin-producing neurons in the thermogenic response by BAT has also been investigated in rats. In line with the aforementioned results, OX-AB rats compared to wild-type rats have a reduced thermogenic response to basic life events, such as light changes and food intake, and after specific stimuli, such as stress or cold (105, 106). These results indicate the involvement of the orexin-producing neurons in the thermogenic regulation by BAT, at least partly regulated by glutamate rather than the orexin neuropeptide itself.

Besides the effect of orexin-producing neurons on BAT activity, orexin-producing neurons are also thought to play a pivotal role in the development and differentiation of BAT early in life. In 2011, Sellayah et al. (107) used an orexin null mouse model, in which mice are orexin-deficient since birth. They observed more rapid weight gain on a high-fat diet and an impaired diet-induced thermogenesis compared to wild-type mice. Morphologically, BAT appeared to be less brown with fewer mitochondria, lipid droplets, and intracellular triglycerides compared to in wild-type mice. White adipose tissue, on the contrary, appeared to be normal. This suggests that orexin influences the differentiation of BAT in the developmental stage. They also showed that restoring orexin during the prenatal stage of the orexin null pups resulted in recovery of BAT morphology in the newborn pups. The effect of orexin on the development and differentiation of BAT in the prenatal phase is suggested to be primarily mediated by OXR1 and not by OXR2 (108). Interestingly, orexin seems to be important not only in the prenatal development of BAT but also in the differentiation of BAT later in life. With increasing age, BAT function declines, and it appears morphologically to be more white (109, 110). Chronic injection (2 weeks) of orexin intraperitoneally in old (2-years-old) mice improved age-related decline in BAT morphology and function. In line with this, orexin-producing neuron ablation caused characteristics of aging in BAT (109). Thus, on top of the thermogenic function of BAT, orexin-producing neurons also appear to play a crucial role in the differentiation and development of BAT, not only in early life but also during aging.

## Neuroendocrine Pathways Involved in Orexigenic Thermoregulation

Several research groups aimed to identify the neuroendocrine pathway involved in orexigenic thermoregulation (Figure 1). Inhibition of the GABAergic tone of neurons in the LHA was shown to increase sympathetic nervous signaling and BAT activity (111). When neurons in the DMH or RPa were inhibited, the effect was reversed. This suggests that at least one of the BAT stimulatory pathways runs via the disinhibition of LHA, which in turn excites the DMH and RPa. However, it remained unclear whether this effect is the result of orexin-producing neurons or other neurons in the LHA (111). In 2011, this pathway was further unraveled by using retrograde anatomical tracing from BAT toward the hypothalamus and the simultaneous immunohistochemical staining of orexin-producing neurons. The researchers demonstrated the existence of orexigenic connections between the perifornical lateral hypothalamus and the rRPa and dense orexin innervation on the rRPa, strongly pointing toward the involvement of orexin in the aforementioned thermogenic pathway (112). It remains to be elucidated what exact effect the orexin-producing neurons have on the rRPa. Proposed mechanisms include a presynaptic effect on glutamate receptors or a rather postsynaptic effect, either direct or by modulating GABA signaling (113). Additionally, other brain regions were shown to be important in thermoregulation by BAT. The cerebral cortex seems to be important in prostaglandin-induced hyperthermia, and an intact VMH appears to be necessary for the activation of thermogenesis (97, 99). Martins et al. (114) demonstrated how the VMH is involved in the orexigenic regulation of BAT thermogenesis. They showed a pathway in which BMP8B, via inhibition of AMPK in the VMH and glutaminergic signaling, results in stimulation of OXR1 in the LHA and subsequently activation of BAT thermogenesis. Interestingly, this pathway was dependent on estrogen and was thus exclusively observed in females (114). This result might be of interest because of the well-known gender differences in fat storage. Women generally exhibit more subcutaneous fat, which has higher potency for lipolysis and browning as compared to visceral fat, which is found to a higher extent in men (115). Gender differences in the presence and metabolic activity of supraclavicular BAT also seem to favor women (115, 116). However, as mentioned before, no gender differences in the prevalence of adiposity in patients with narcolepsy type 1 have been reported. The pathway involving inhibition of AMKP in the VMH connects nicely with the aforementioned reports from other research groups describing the involvement of the LHA in the sympathetic stimulation of BAT. Recently, another interesting pathway involving the brain dopamine system has been described. The dopamine receptor 2 on GABAergic neurons in the LHA and zona incerta appears to be able to upregulate *Ucp1*, BAT thermogenesis, and energy expenditure in mice. This pathway is dependent on orexin signaling (117). The fact that dopamine plays a role in the orexigenic thermogenesis was reported before, when ablating the dopamine neurons resulted in abolishment of the orexigenic increase in body temperature (98).

Altogether, the majority of rodent studies point toward a crucial role for orexin-producing neurons rather than the orexin neuropeptides in BAT functionality. On top of this, orexin neuropeptides appear to play a role in BAT development and differentiation.

## Human Perspective

The emerging evidence that the orexin system is involved in BAT function in rodents leads to the hypothesis that impaired BAT functionality could be causally involved in the increased adiposity in patients with narcolepsy type 1. To the best of our knowledge, only two recent studies have investigated this issue. One study investigated BAT activity between patients with narcolepsy type 1 and healthy controls. In all subjects, [^18^F]FDG uptake by BAT was measured after 2 h of mild cold exposure. No difference was found between the groups, indicating no difference in glucose uptake by BAT. However, there are several limitations imposed by the study design. This study was performed with a small sample size (*n* = 7 per group), and a fixed mild temperature was used as a cold stimulus to activate BAT. Possibly, this does not result in maximal activation of BAT for all individuals. Especially for patients with narcolepsy type 1, who are reported to have a higher distal skin temperature, probably due to vasodilatation, cold exposure could have evoked a larger stimulus on BAT, leading to overestimation of their BAT activity compared to the healthy controls (118). In addition, the use of [^18^F]FDG-PET/CT scans to visualize human BAT is currently under debate, considering that BAT mainly combusts triglyceride-derived fatty acids, while [^18^F]FDG-PET/CT scans only visualize glucose uptake. On top of that, glucose uptake is highly impaired in insulin-resistant conditions, which could be an issue for measuring BAT in overweight and obese people (119). However, a valuable addition in this study was the use of [^123^I]MIBG-SPECT/CT scanning, which visualizes sympathetic nervous stimulation. They showed intact adrenergic innervation in the BAT of patients with narcolepsy type 1, suggesting that at least the sympathetic outflow is maintained (120). In the same year, Drissi et al. (121) aimed to investigate adipose tissue distribution measured with MRI in a thermoneutral condition. They showed no differences in supraclavicular BAT between patients with narcolepsy type 1 and healthy controls. It is important to note that this is in line with preclinical studies showing that BAT in mice appeared to be morphologically normal in OX-AB mice. However, BAT functionality was attenuated in the absence of the orexin-producing neurons (104), and BAT functionality was not examined in the study of Drissi et al. (121).

The limitations of the available human studies illustrate the challenge of translating preclinical studies into human studies. Different animal models exist that mimic the phenotype of narcolepsy type 1. However, none of the currently existing models adequately and precisely reflect the human situation. As mentioned above, in patients with narcolepsy type 1, orexin-producing neurons are lost, resulting in the loss of orexin but also of the co-expressed glutamate, dynorphin, and NARP (25). Therefore, mouse models that mimic narcolepsy type 1 by means of inhibition of the OXR or a lack of prepro-orexin might not be fully adequate. Orexin-producing neuron ablation appears to come closer to the human phenotype (35). Nonetheless, patients with narcolepsy type 1 develop the disease during life following an autoimmune response, which hinders the development of escape pathways to substitute for the lost functions. Accordingly, a mouse model has been developed that uses a tetracycline-controlled transcriptional activation system and is able to time the orexin-producing neuron degeneration (122). This mouse model strongly resembles the human situation and could therefore provide more valuable information about metabolic changes that occur after the onset of narcolepsy type 1.

## Conclusion and Future Perspectives

Patients with narcolepsy type 1 are at increased risk for obesity, despite a normal to reduced food intake. Results from preclinical studies strongly suggest that destruction of orexin-producing neurons leads to diminished BAT functionality and subsequently to impaired energy homeostasis. Preclinical studies revealed several neuroendocrine pathways involved in orexigenic thermoregulation. However, to date, convincing evidence from human studies about impaired BAT function in patients with narcolepsy type 1 is lacking. Currently, new techniques for visualization and quantification of BAT volume and activity are evolving at a high rate, including the use of magnetic resonance imaging to measure fat fraction and perfusion changes in BAT upon activation. This could be promising for patient populations in which detecting BAT activity by [^18^F]FDG-PET/CT scan is hampered due to insulin resistance. A crucial influencing factor to take into account is a different temperature perception in patients with narcolepsy type 1, which can markedly influence the cold-induced measurements often used in BAT research. In addition, more insight is needed into the involvement of the orexin-producing neurons in neuroendocrine pathways involved in energy expenditure by BAT and whether these pathways are disturbed in narcolepsy type 1 patients. To further explore the contribution of skeletal muscle and white adipose tissue in the regulation of energy metabolism after orexin deficiency, tissue biopsies in combination with *ex vivo* mitochondrial respiration measurements and metabolomics could provide additional knowledge on metabolic processes within these tissues. Although no significant gender differences in obesity rates amongst patients with narcolepsy type 1 have yet been described, the involvement of gender hormones in the characteristics of both WAT and BAT and their central actions in the brain raise questions about whether sex hormones influence the development of adiposity after orexin-producing neuron destruction. Therefore, the influence of gender is a topic that requires further study.

More knowledge about the pathogenesis underlying the increased BMI in patients with narcolepsy type 1 could lead to the development of more effective treatment options to counter their increased adiposity and to improve their metabolic health. Therefore, additional studies are highly warranted to further investigate BAT functionality in this metabolically compromised patient population.

## Author Contributions

MES wrote and submitted the manuscript. MSS, RF, GL, PR, and MB critically reviewed the manuscript.

### Conflict of Interest

The authors declare that the research was conducted in the absence of any commercial or financial relationships that could be construed as a potential conflict of interest.
